# List of Reviewers

**DOI:** 10.1093/jrr/rrae093

**Published:** 2024-12-04

We would like to express our greatest appreciation to our reviewers. *Journal of Radiation Research* Vol. 65 (2024) benefited from the comments and insights from the following 280 reviewers. We sincerely thank them for their time devoted and effort which contributed to the improvement of the journal (Reviewers who reviewed three or more manuscripts are listed in bold font.)

Takanori Adachi

Rihito Aizawa

Keiichi Akahane

Makoto Akashi

Kouhei Akazawa

Tetsuo Akimoto

Ken Ando

**Yusuke Anetai**

Kentaro Ariyoshi

Molykutty Aryankalayil

Friedrich Boege

William Chen

Augustine E. Chen

Jing Chen

Simon Cheng

**James Chow**

Matt Coleman

Indra Das

Michael De Lisio

Takeshi Ebara

Stephen Egbert

Felix Ehret

**Yasuo Ejima**

Michael Freeman

Yukio Fujita

Manabu Fukumoto

Hisanori Fukunaga

Yukihiro Furusawa

Akihiro Haga

Kanya Hamasaki

Takashi Hanada

Kiyofumi Haneda

Hideyuki Harada

Hiroshi Harada

Yoshihiro Hasegawa

Arifumi Hasegawa

Takayuki Hashimoto

**Masaharu Hata**

Tomonori Hayashi

Tom Hei

Tomoo Hidaka

Yukihito Higashi

Hideaki Hirashima

Takero Hirata

Ryoichi Hirayama

Nobuyuki Hirohashi

Tokuhisa Hirouchi

Masahiro Hosoda

Roger Howell

Naonori Hu

Hiroshi Igaki

Noriko Ii

Hitoshi Ikushima

Hiromasa Imaizumi

Nobuki Imano

Tatsuhiko Imaoka

Koji Inaba

Ken Inoue

Masayori Ishikawa

Junya Ishikawa

Hiromichi Ishiyama

**Fumiaki Isohashi**

Satoshi Itasaka

Atsushi Ito

Hiroyuki Itoh

Hiromitsu Iwata

**Keiichi Jingu**

Noriyuki Kadoya

Michiaki Kai

**Atsushi Kaida**

Tomohiro Kajikawa

Naoya Kakimoto

Ryo Kakino

Nobuyuki Kanematsu

Yuko Kaneyasu

Koki Kasamatsu

Ikuo Kashiwakura

Atsuto Katano

Takahiro Kataoka

Atkins Katelyn

Shingo Kato

Norio Katoh

Daisuke Kawahara

**Hidemasa Kawamura**

**Shinji Kawamura**

Mariko Kawamura

**Takashi Kawanaka**

Masahiro Kenjo

Tomoki Kimura

Rumiko Kinoshita

Noriko Kishi

Kazuki Kitabatake

Satoshi Kito

Yusuke Koba

**Kazuma Kobayashi**

Satoshi Kodaira

Takeshi Kodaira

Akito Koganezawa

Masaoki Kohzaki

Takahumi Komiyama

Tatsuya Konishi

Masashi Koto

Munehiko Kowatari

Nobuteru Kubo

**Hiroaki Kumada**

Satoshi Kumagai

Jun Kumagai

Kyo Kume

Chie Kurokawa

**Masahiko Kurooka**

Xiaoying Liang

Qiang Liu

Ning-Ang Liu

Dengqun Liu

Katsuya Maebayashi

Munetoshi Maeda

Yoshikazu Maeda

Taiki Magome

Kailash Manda

Takamitsu Masuda

Akihiko Masuda

Koji Masui

**Hiroaki Matsubara**

**Yoshitaka Matsumoto**

Yoshihisa Matsumoto

Masayuki Matsuo

Shinya Matsuura

Yoichrou Matuo

Hiroshi Mayahara

Hiroshi Mitani

Yasuo Miura

Hideharu Miura

Masahiko Miura

**Naoki Miyamoto**

Yasushi Miyazaki

Masashi Mizumoto

Hideyuki Mizuno

Hajime Monzen

Shinichiro Mori

Shimizu Morihito

Akinori Morita

Naoya Murakami

Yuji Murakami

Masashi Murakami

Michio Murakami

Kazutoshi Murata

Masaki Nagane

Takahiro Nakamoto

**Mitsuhiro Nakamura**

**Kiyonao Nakamura**

Asako Nakamura

Katsumasa Nakamura

Satoaki Nakamura

Satoshi Nakamura

Ikuo Nakanishi

Hidetsugu Nakayama

Ikuno Nishibuchi

Kentaro Nishioka

Akihiro Nohtomi

Eka Nugraha

Sae Ochi

**Toshiyuki Ogata**

Kazuhiko Ogawa

Etsuyo Ogo

Shingo Ohira

Tatsuya Ohno

Megu Ohtaki

Masataka Oita

**Masahiko Okamoto**

Hiroyuki Okamoto

Shohei Okazaki

**Noriyuki Okonogi**

Rikiya Onimaru

**Tomohiro Ono**

**Kaoru Ono**

Yuka Ono

Takashi Ono

Seiichi Ota

**Yuki Otani**

Natsuo Oya

Igor Pshenichnov

Masahide Saito

Katsuyuki Sakanaka

Tetsuya Sakashita

**Ritsu Sakata**

**Vasanthan Sakthivel**

Takayuki Sakurai

Hideyuki Sakurai

Makoto Sasaki

Megumi Sasatani

Katsuya Sato

Qi Sharon

Keiko Shibuya

Naoto Shikama

Naoya Shikazono

Mikio Shimada

Tsutomu Shimura

Katsuyuki Shirai

Hiroki Shirato

**Elena Shishkina**

Shyam K. Shrivastava

Igor Shuryak

Toshinori Soejima

Masanori Someya

**Valeriy Stepanenko**

Hiromi Sugiyama

Lue Sun

Masaaki Sunaoshi

**Keiji Suzuki**

Minoru Suzuki

Osamu Suzuki

Ryo Suzuki

Yoshiyuki Suzuki

Gen Suzuki

Hirotaka Tachibana

Hidenobu Tachibana

Senzo Taguchi

Masashi Takada

Kenta Takada

Noriko Takagi

Masaru Takagi

Kenji Takahashi

Takeo Takahashi

Toru Takakura

Shigeyuki Takamatsu

Noboru Takamura

Seishin Takao

Kazuya Takeda

Hideki Takegawa

**Ariga Takuro**

Tomoaki Tamaki

Yuki Tamakuma

**Satoshi Tanabe**

Kenichi Tanaka

Hiroki Tanaka

Hideo Tatsuzaki

Hiroaki Terato

Toshiyuki Terunuma

Naoki Tohyama

Sunao Tokumaru

Yuki Tominaga

Masanori Tomita

Natsuo Tomita

Shin Toyoda

Masataka Tsuda

Kouki Uchinomiya

Rei Umezawa

Takashi Uno

Megumi Uto

**Satoru Utsunomiya**

**Yuki Wada**

Masaru Wakatsuki

Richard Wakeford

Hong-Jian (James) Wei

Philip Wu

Wei Xiong

Masashi Yagi

Ichiro Yamaguchi

Masaru Yamaguchi

Kentaro Yamamoto

Shinichi Yamashita

Motohiro Yamauchi

Chikako Yamauchi

Koichi Yasuda

Hiroshi Yasuda

Keisuke Yasui

Kenji Yoshida

Ken Yoshida

Yukari Yoshida

Yuya Yoshimoto

Ryoichi Yoshimura

Michio Yoshimura

Shinji Yoshinaga

**Hironori Yoshino**

Yasuo Yoshioka

Shuyu Zhang

Yawei Zhang

Chi Zhang

Lei Zhao

Dejun Zhou

